# Bronchoscope-Guided Airway Rescue via an I-gel™ for Haematoma-Induced Airway Obstruction Following Anterior Cervical Spine Surgery

**DOI:** 10.7759/cureus.34990

**Published:** 2023-02-14

**Authors:** Philip L Stagg

**Affiliations:** 1 Faculty of Health Sciences and Medicine, Bond University, Gold Coast, AUS

**Keywords:** can’t intubate can’t oxygenate, bronchoscope-guided tracheal intubation, supraglottic airway device (sad), difficult airway society (das), anterior cervical discectomy and fusion (acdf)

## Abstract

Airway obstruction requiring emergency airway rescue is an uncommon yet potentially fatal complication following anterior cervical discectomy and fusion. This report describes rapid clinical deterioration after anterior cervical discectomy and fusion despite haematoma evacuation. After failing to secure the airway with awake bronchoscope-guided tracheal intubation and video-laryngoscopy, an I-gel™ supraglottic airway was inserted, and alveolar oxygenation was restored. Bronchoscope-guided intubation was easily achieved via the I-gel™ lumen. The practicality of this technique for airway rescue in the context of a high-stakes time-critical airway emergency is discussed.

## Introduction

Wound haematoma causing airway obstruction after anterior cervical surgery has a reported incidence of 0.2-1.9% [1]. This potentially life-threatening clinical scenario requires specialised knowledge and advanced airway management skills. Most events occur within 48 hours after surgery, and reintubation is frequently required [2]. Deaths due to airway obstruction have occurred [2]. 

Guidelines exist for the management of post-operative neck haematoma [1,3,4]. However, heterogeneity exists between these guidelines, particularly regarding aspects such as task sequencing and nuances of haematoma evacuation and airway management. Furthermore, guidelines often leave the final airway management technique to the end user, as the burden or responsibility will ultimately be context- and user-dependent. As such, there is a significant scope to learn from specific cases where context-dependant airway strategies have led to positive outcomes in this time-critical high-stakes scenario. 

In this case, a rapidly expanding neck haematoma following anterior cervical discectomy and fusion (ACDF) caused a life-threatening airway compromise. Airway obstruction progressed despite prompt haematoma evacuation, and initial intubation attempts were unsuccessful. Airway rescue was achieved via bronchoscope-guided tracheal intubation using the I-gel™ lumen. The strengths and weaknesses of using this technique for airway rescue during a high-stakes time-critical airway emergency are discussed, including areas for improvement.

## Case presentation

A 91-kilogram, 60-year-old male presenting with cervical myelopathy was scheduled for ACDF. His past medical history was significant for obstructive sleep apnoea, asthma, and a cerebrovascular accident (CVA). He had fully recovered from the CVA. Medications included atorvastatin 40 mg and aspirin 100 mg. Aspirin was withheld for one week prior to surgery. 

Anaesthesia was induced and maintained, using propofol and remifentanil total intravenous anaesthesia. Neuromuscular blockade was achieved with vecuronium 10 mg. The trachea was intubated with an 8.0 internal diameter (ID) tube, and direct laryngoscopy revealed a grade-one Cormack-Lehane glottic view. The surgical procedure was performed uneventfully, at the C4/5 level, via a right-sided transverse incision. Neuromuscular block was reversed with sugammadex 200 mg. 

Two hours after surgery, while in the neurosurgical ward, the patient became agitated. He stated that he needed to keep clearing his throat and wanted to cough. He was noted to have vocal changes and a small amount of bleeding through the dressing. He became tachycardic, tachypnoeic, and hypertensive. The nursing staff called the operating theatre and requested that the surgeon or anaesthetist review the patient. Being unable to attend while operating, the surgeon requested an urgent review by the intensive care specialist in the post-anaesthetic care unit (PACU). 

The patient arrived in the PACU sitting upright, with 10 L/min oxygen delivered via nasal prongs. He was now visibly distressed, having rapidly deteriorated during the transfer. His arterial oxygen saturation was 91%. The intensive care specialist arrived, promptly removed the sutures, and evacuated the haematoma. Despite brief improvement, the patient remained visibly distressed with persistent hypoxia. The difficult intubation trolley was obtained, and the first anaesthetist to arrive attempted awake tracheal intubation (ATI) using a 4.0 mm diameter bronchoscope (Karl Storz, Tuttlingen, Baden-Württemberg, Germany). This procedure was conducted without local anaesthesia, given the patient's increasingly obtunded state. After one unsuccessful attempt, this procedure was aborted. The patient’s oxygen saturation fell to 88%. Multiple actions then occurred in parallel. 100% oxygen was delivered via a self-inflating bag-valve mask in the seated position. Intubation equipment, including a video laryngoscope, and emergency front of neck access (FONA) were organised. Attempts were made to obtain an otolaryngologist for assistance. The treating surgeon arrived and requested urgent access to the neck. 

While the surgeon quickly investigated the neck wound, the patient became unconscious with a completely obstructed airway. Arterial oxygen saturation fell to 39%. Cardiorespiratory arrest was imminent, although chest compressions were not required. A CMAC® D-Blade (Karl Storz, Tuttlingen, Baden-Württemberg, Germany) video laryngoscope was inserted, to attempt tracheal intubation. During laryngoscopy, high-flow oxygen therapy was administered via the nares. Copious secretions and distorted anatomy were encountered, and a view of the glottis was not achieved. The intubation attempt was aborted. Based on patient weight (> 90 kg) and with a view to subsequently passing a tracheal tube through the device, a size 5 I-gel™ (Intersurgical®, Wokingham, Berkshire, United Kingdom) was inserted and attached to the bag-valve mask. Assisted ventilation was commenced successfully, and arterial oxygen saturation rapidly returned to 98%. 

**Figure 1:**
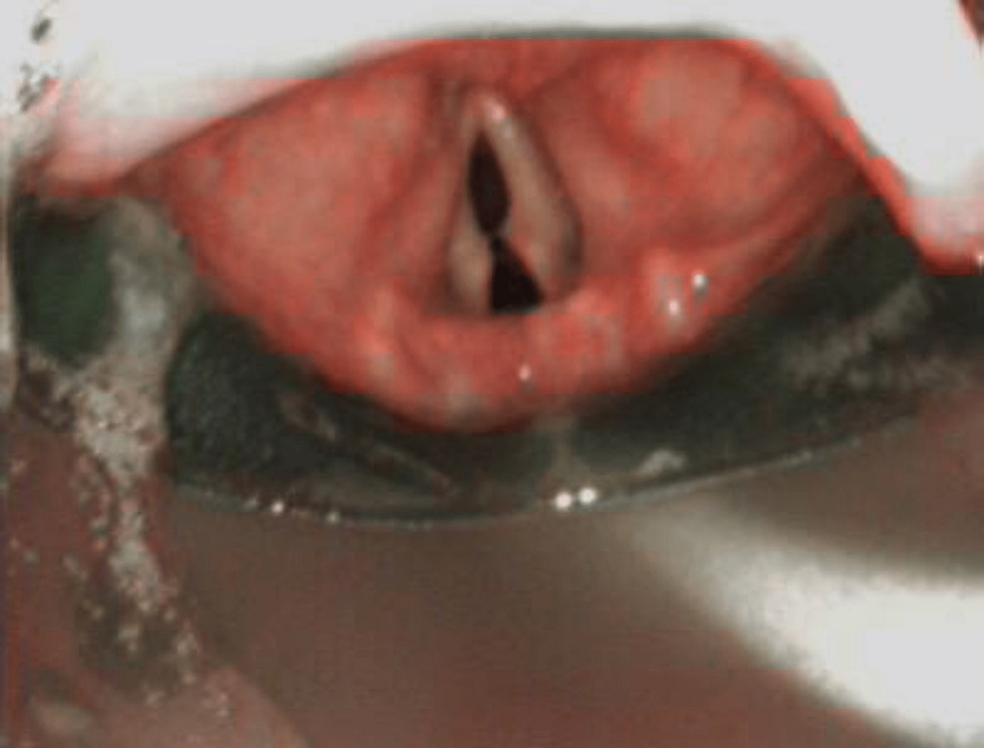
View of glottic structures via an I-gel™ 5 supraglottic airway using a 4.0 mm diameter bronchoscope.

**Figure 2:**
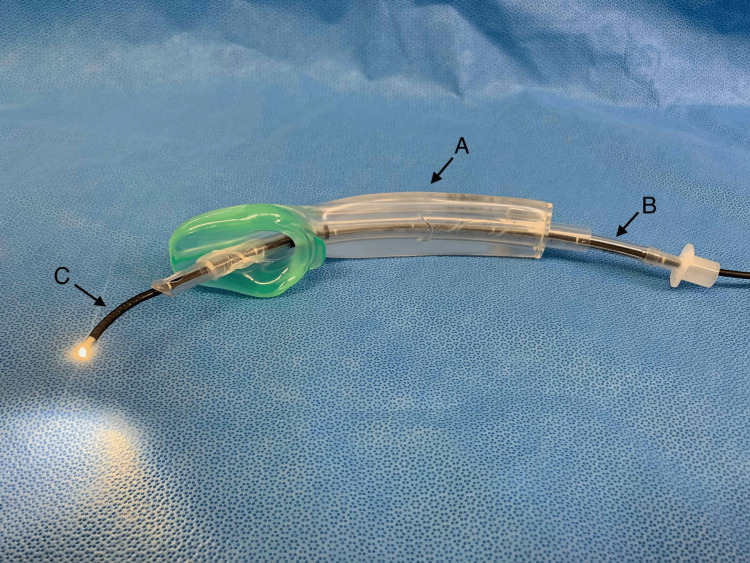
Equipment used during tracheal intubation via the I-gel™. (A) A size 5 I-gel™. (B) A well-lubricated size 7.0 mm PVC tracheal tube, inserted into the lumen of the I-gel™. Note that the cuff has been well lubricated and fully deflated to ensure ease of insertion. (C) A 4.0 mm bronchoscope inserted into the tracheal tube lumen for bronchoscope-guided intubation.

During re-oxygenation, the treating team discussed management options. Midazolam 5 mg was administered. A well-lubricated 7.0 ID tracheal tube with a fully deflated pilot cuff was then placed over the bronchoscope and inserted into the ventilation lumen of the I-gel™ (Figure 1). Three millilitres of 1% lignocaine were delivered onto the vocal cords via the bronchoscope working channel. The bronchoscope was then guided into the trachea and the tracheal tube advanced into the trachea. On withdrawal of the bronchoscope, the tracheal tube tip was noted to be appropriately located above the carina. The tracheal tube was connected to the bag-valve mask and assisted ventilation commenced. Capnography confirmed correct tube placement. The tracheal tube was visualised, through the transparent I-gel™ wall, to be at an appropriate depth of 24 cm at the teeth. During tracheal intubation, approximately 50 mg of propofol was administered. Oxygen saturation was maintained throughout. The patient was transferred back to the operating room, with both the I-gel™ and tracheal tube in situ, due to the perceived risk of dislodging the tracheal tube if an attempt to remove the I-gel™ was made at that time. Figure 2 depicts the equipment used during tracheal intubation via the I-gel™. 

**Figure 3:**
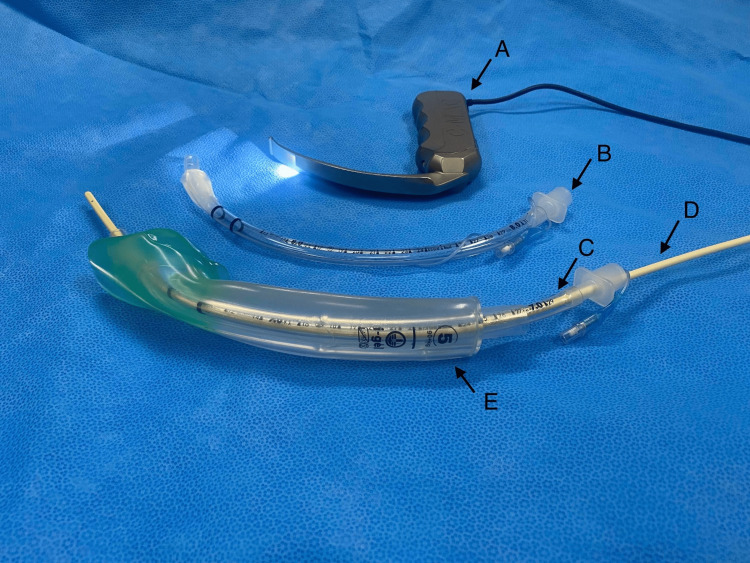
Equipment used for airway exchange. (A) A Karl Storz CMAC® 4 Blade. (B) An 8.0 mm PVC tracheal tube. (C) A 7.0 mm tracheal tube. (D) A 14 Fr 83 cm Cook® airway exchange catheter. (E) A size 5 I-gel™. The I-gel™ and 7.0 mm tracheal tube were removed as a single unit over the exchange catheter under direct vision using the CMAC® 4 Blade. The 8.0 mm tube was successfully inserted under direct vision into the trachea over the exchange catheter.

Upon return to theatre, neuromuscular blockade was achieved with rocuronium 100 mg and anaesthesia was maintained on sevoflurane. The surgeon successfully evacuated the remaining haematoma and obtained adequate haemostasis. At the end of the procedure, the anaesthetic team exchanged the tracheal tube. A 14 Fr 83 cm Cook® airway exchange catheter (Bloomington, Indiana, United States) was inserted. The I-gel™ and tracheal tube were removed together. A CMAC® 4 blade was inserted into the pharyngeal cavity. A full view of the glottis was obtained on the CMAC screen. An 8.0 mm ID tracheal tube was inserted easily on first pass. Figure 3 depicts the equipment used for the airway exchange. 

In intensive care, extubation was delayed due to ongoing swelling and poor views of the glottis with conventional direct laryngoscopy. Intravenous dexamethasone 4mg was administered three times a day to treat this. Extubation delays were further compounded by Klebsiella ventilator-associated pneumonia (managed successfully with piperacillin/tazobactam), and confusion on daily weens. He was successfully extubated on day six and returned to the neurosurgical ward on day seven. 

**Figure 4:**
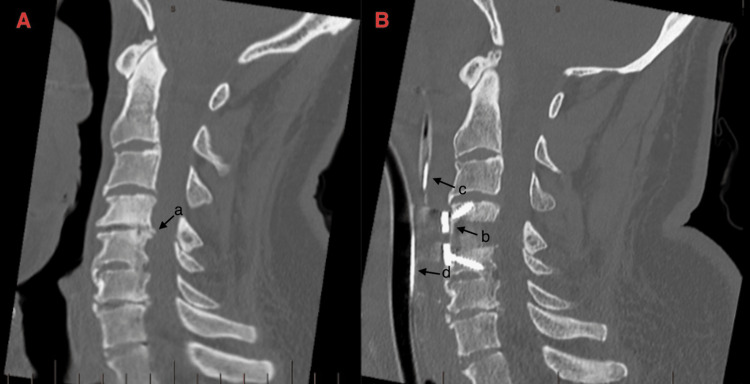
Pre-operative and post-operative computed tomography scans. (A) Pre-operative computed tomography (CT) scan of the cervical spine demonstrating multilevel degenerative changes. (a) At the C4/5 level, there is a disc osteophytic bulge, with posterior osteophytic protrusion, causing mild to moderate impingement on the anterior chord. (B) Post-operative CT. (b) C4/5 anterior discectomy and fusion after successful haematoma evacuation. Anatomical alignment is maintained, with degenerative changes in the adjacent levels. (c) Nasogastric tube. (d) Tracheal tube.

Figure 4 displays the cervical spine both pre-operatively and post-operatively after the haematoma was evacuated.

Outcome and follow-up 

Anaesthetic review on day 10 found the patient in high spirits and happy with the improvement in his cervical myelopathy symptoms. He had no recollection of the events that transpired. He was discharged on day 13 to a rehabilitation facility. 

Six weeks after surgery, the patient wrote to express his gratitude and had made a full recovery. 

## Discussion

This report describes the prompt recognition and management of life-threatening upper airway obstruction after ACDF. While initial attempts to restore the airway were unsuccessful, airway rescue was successfully achieved via bronchoscope-guided tracheal intubation via the I-gel™ lumen. The Difficult Airway Society (DAS) guideline for the unanticipated difficult airway recommends that, after failed intubation, the primary goal should be the maintenance and restoration of alveolar oxygenation via the insertion of a second-generation supraglottic airway device (SAD) [5]. Successful SAD insertion and reoxygenation provide practitioners time to “stop and think” and assess their options. These options include waking the patient, performing the surgical procedure with the SAD, converting the SAD to a tracheal tube or surgical airway, or replacing the airway device [5]. In non-urgent situations, waking the patient should be the primary consideration. However, in this case, it was necessary to urgently return to the operating theatre given the evolving emergency. Furthermore, there was a tremendous risk of repeat complete airway obstruction without a definitive airway. The DAS guideline discusses intubation via a SAD as one of the several options, but only when the situation is deemed appropriate [5]. Blind intubation attempts via a SAD are, however, not recommended. Success rates of greater than 90% have been demonstrated for fibreoptic tracheal intubation via the I-gel™, with equivalence to the Laryngeal Mask Airway (LMA®) Fastrach™ (Teleflex®, Athlone, Westmeath, Ireland) [6,7]. Fibreoptic glottic visualisation via the I-gel™ is significantly better than with the LMA® Fastrach™ [7] and the LMA® Supreme™(Teleflex®) [8], with less epiglottic downfolding. The I-gel™ user guide [9] recommends the following maximum size of the tracheal tube when intubating via the device: I-gel™ 3 - 6.0 mm tracheal tube; I-gel™ 4 - 7.0 mm tracheal tube; I-gel™ 5 - 8.0 mm tracheal tube. Despite using a size 5 I-gel™ here, a slightly smaller 7.0 mm tracheal tube was chosen to minimise factors that might impede first-pass intubation success, such as excessive friction when passing the tracheal tube through the I-gel™ lumen, or tracheal tube impingement on laryngeal structures due to a size difference between the bronchoscope and a larger tracheal tube.

A potential limitation of choosing direct intubation via the I-gel™ lumen was the risk of inadvertently dislodging the tracheal tube while removing the I-gel™. This risk acquired greater significance given the airway emergency. Given this, it was elected to return to the operating theatre without attempting I-gel™ removal. Alternatively, an Aintree Intubation Catheter™ (COOK®, Bloomington, Indiana, USA) could have been used to achieve tracheal intubation and was considered. Indeed, high tracheal intubation success rates via a SAD have been observed using an Aintree Intubation Catheter™ [10]. However, no trials have compared direct fibreoptic tracheal intubation to Aintree Catheter™ for facilitating intubation via the I-gel™. Both techniques have a low failure rate. Advantages of choosing direct intubation through the I-gel™ in this case included not having to remove the device by which oxygenation was restored and a reduced number of procedural steps. Reducing procedural steps was considered beneficial, with only limited cognitive bandwidth and an unstable patient with reduced oxygen reserve. Furthermore, a full armamentarium of adjuncts such as the Aintree Catheter™ may not be available in a rapidly evolving emergency, and a bronchoscope with an external diameter of less than 48mm is required to use the Aintree Catheter™ which, while available here, may be unavailable at short notice. Additional disadvantages of direct intubation via the I-gel™ include the lack of a proprietary stabiliser rod to help facilitate I-gel™ removal and a smaller tracheal tube being required to intubate directly through the I-gel™. Notwithstanding these limitations, studies have demonstrated no difficulty in removing the I-gel™ after using it as an intubation conduit. Removal options include using the LMA® Fastrach™ stabiliser rod [6], Magill's forceps [11], or attaching a tracheal tube one size smaller to the connector end of the in situ tube as a stabiliser [12]. In this case, we decided not to remove the I-gel™ prior to returning to the operating theatre, due to the critical state of the airway, the suboptimal management location (i.e., the recovery room), and the risk of cognitive strain impeding performance. 

**Figure 5:**
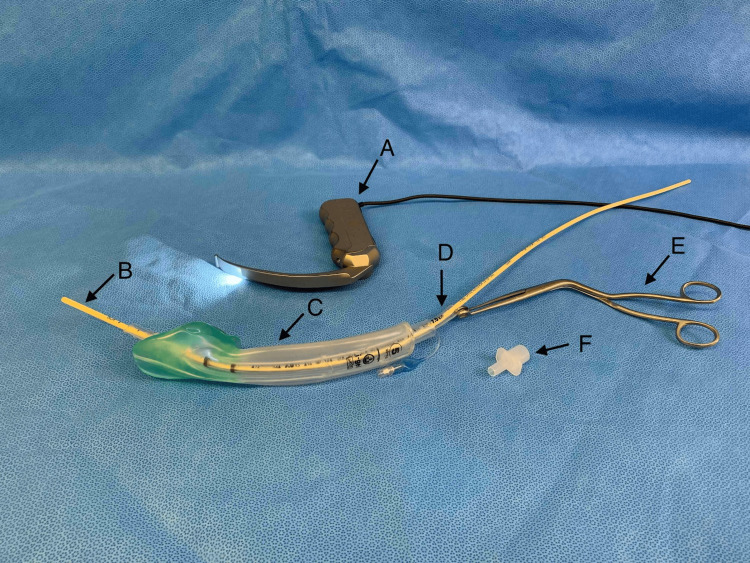
Proposed method of removing an I-gel™ after tracheal intubation in a high-stakes airway emergency. (A) A videolaryngoscope. (B) An airway exchange catheter. (C) An I-gel™. (D) A tracheal tube with 15 mm universal connector removed. (E) Magill forceps. (F) The 15 mm connector from the tracheal tube. After inserting the airway exchange catheter into the patient's airway via the tracheal tube, Magill forceps are used as a stabiliser rod to hold the tube in place while the I-gel™ is carefully removed. Once the I-gel™ cuff has entered the oropharynx, the tracheal tube can then be grasped within the mouth while the I-gel™ is completely removed. The presence of the in situ exchange catheter and a nearby videolaryngoscope provides an added margin of safety if the tracheal tube is inadvertently dislodged. Paediatric Magill forceps may be required to fit within the lumen of a smaller sized I-gel™.

After formal wound exploration, the I-gel™ and tracheal tube were removed and replaced with a larger tracheal tube using videolaryngoscopy and an airway exchange catheter rather than solely removing the I-gel™. By this stage, the patient was stable and staff more relaxed. This more controlled environment, along with some time having elapsed since the airway emergency, potentially afforded a psychological advantage in the performance of this step. Further potential advantages of formal tracheal tube exchange at this later point were the ability to grade the airway after formal haematoma evacuation and the opportunity to insert a larger tracheal tube than was possible via the I-gel™ prior to transfer to the intensive care unit. A disadvantage was the requirement to remove a definitive airway, albeit with the added protection of the airway exchange catheter. On reflection, a potentially safer option could have been to first insert an airway exchange catheter into the trachea prior to using a pair of Magill’s forceps as a stabiliser to keep the original tracheal tube in situ (Figure 5). This method would obviate the need for any formal tracheal tube exchange while facilitating removal of the I-gel™ using a recognised technique, as well as affording an additional margin of safety with the presence of the in situ exchange catheter, in the event that the tracheal tube was dislodged. If the event that the tracheal tube was partially dislodged during SAD removal, it could be reinserted over the exchange catheter in conjunction with videolaryngoscopy. This potentially safer hybrid method could prove useful in high-stakes airway emergencies, where every effort should be made to prevent tracheal tube dislodgement and minimise risk. Further studies are required to elucidate the benefits of such a technique. 

While this report describes successful tracheal intubation via an I-gel™, multiple airway management techniques have been successful for neck haematoma, before or after induction of general anaesthesia [1,13-15]. In this case, awake tracheal intubation was unsuccessful. However, awake tracheal intubation with a flexible bronchoscope is often a safe and appropriate initial airway management technique and should be considered [1,3,13,14]. Awake tracheal intubation was successful in 15 out of 20 (75%) cases of anterior neck haematoma [13]. Notwithstanding, practitioners should remain cognisant that despite its many benefits, awake tracheal intubation may be significantly more difficult in critically unwell patients [16]. Awake tracheal intubation with a video laryngoscope is another option and has a similar reported success rate compared to the fibreoptic bronchoscope in controlled scenarios [17]. This technique is reported to be potentially beneficial in patients with secretions in the airway. However, it is unlikely to be tolerated or ergonomic in cases of neck haematoma, as these patients should remain upright while conscious to prevent worsening of their airway obstruction. ATI via a SAD affords similar benefits and high success rates in elective difficult airway scenarios [11,17]. ATI via a SAD has many similar features to the ultimately successful technique used in this case, however, with several caveats. True ATI via a SAD requires ample time to topicalise the airway and a very cooperative patient; neither were present in this case. However, once the SAD had been inserted, we did observe that bronchoscope visualisation of the larynx was excellent and tracheal intubation was easily achieved with the I-gel™ providing a clear and unencumbered passage directly to the vocal cords, requiring minimal bronchoscope-manoeuvring dexterity in this high-stress situation. Bronchoscope-guided intubation via a SAD has previously been given the moniker the “low-skill fibreoptic intubation” [18], making it a potentially easier option for those less experienced. To this sentiment, I posit that the simplicity of bronchoscope-guided intubation via a SAD also lends itself to high-stress time-critical emergencies where the airway must be definitively secured, and a SAD has already been inserted successfully. 

In this case, the failure of prompt haematoma evacuation to stabilise the airway or help facilitate initial intubation attempts is worthy of discussion. Indeed, conflicting views still exist about the benefits, timing, and personnel most suited to performing haematoma evacuation. Anecdotally, from personal discussions with surgeons performing neck surgery, some are still reluctant for first responders to perform emergency haematoma evacuation. Reluctance stems from fear that inexperienced staff will cause further damage, fail to open all investing layers, and the belief that haematoma evacuation will not resolve the glottic oedema that is responsible for airway obstruction [3]. Some authors suggest that haematoma evacuation only be performed after discussion with the treating surgeon [14]. Others propose a single tracheal intubation attempt, after which haematoma is evacuated prior to further attempts [1]. Notwithstanding these views, up-to-date multidisciplinary consensus guidelines overwhelmingly recommend that haematoma evacuations occur early, and prior to tracheal intubation if there are signs of airway compromise [3,4]. All investing layers should be opened and haematoma evacuated [3,4]. This should increase the likelihood of successful intubation [3]. Indeed, in a retrospective analysis of neck haematomas requiring airway management, haematoma evacuation after failed attempts at tracheal intubation facilitated subsequent success in three out of four cases [13]. These guidelines emphasise empowering junior medical and nursing staff to act. All staff involved in the care of patients who have had neck surgery should have regular training in haematoma evacuation [3,4]. While these policies have mainly been developed for post-thyroidectomy care, they should be extended to all patients who have had neck surgery. A post-surgical emergency box, holding all the necessary equipment for haematoma evacuation, is further recommended [3]. 

A potential limitation of the airway management plan presented in this case was that neuromuscular blockade was not administered. Guidelines emphasise the importance of adequate neuromuscular block during difficult airway management [5,16,17]. It is possible that tracheal intubation may have been achieved earlier, during the videolaryngoscopy attempt, had the patient received neuromuscular blockade. This argument is strengthened by the fact that 100% of glottic opening was observed with videolaryngoscopy during tracheal tube exchange, albeit after surgical haemostasis and haematoma evacuation had occurred. Reasons that neuromuscular blockade was not administered included uncertainty regarding equipment availability in the PACU, doubt surrounding the effectiveness of neuromuscular blockade shortly after sugammadex administration, and concerns of an impending can’t intubate, can’t oxygenate scenario. Immediate front of neck access was not readily available, as the surgeon was fixated on identifying the source of bleeding and situationally unaware of the need for cohesive team-focus on the airway emergency. Preliminary studies suggest that when 1.2 mg/kg of rocuronium is administered 30 minutes after 4 mg/kg sugammadex neuromuscular blockade will occur within 2 minutes [19]. Suxamethonium is an obvious alternative if rapid neuromuscular blockade is required shortly after sugammadex administration. Clearly, practitioners should not hesitate to readminister neuromuscular blocking agents if initial attempts at restoring oxygenation fail.

## Conclusions

This case highlights a number of pertinent learning points. First, airway rescue using bronchoscope-guided tracheal intubation via the I-gel™ lumen should be considered by practitioners facing time-critical high-stakes airway emergencies where initial intubation attempts have failed. In this case, it quickly restored airway patency and oxygenation and provided a secretion-free and easily navigated conduit for subsequent fibreoptic intubation requiring minimal bronchoscope-manoeuvring dexterity in a high-stress environment. Second, bronchoscope-guided intubation via an I-gel™ may negate the need for neuromuscular blockade if situation-specific concerns exist; however, neuromuscular blockade should not be withheld if further attempts at restoring oxygenation fail. Finally, current guidelines support the notion that haematoma should be evacuated early by trained personnel if there are signs of airway compromise as this may improve intubation conditions. An emergency pack containing the necessary equipment should be kept with the patient.
